# Hypoxic Preconditioning with Cobalt of Bone Marrow Mesenchymal Stem Cells Improves Cell Migration and Enhances Therapy for Treatment of Ischemic Acute Kidney Injury

**DOI:** 10.1371/journal.pone.0062703

**Published:** 2013-05-09

**Authors:** Xiaofang Yu, Chunlai Lu, Hong Liu, Shengxiang Rao, Jieru Cai, Shaopeng Liu, Alison J. Kriegel, Andrew S. Greene, Minyu Liang, Xiaoqiang Ding

**Affiliations:** 1 Department of Nephrology, Zhongshan Hospital, Shanghai Medical College, Fudan University, Shanghai, China; 2 Department of Thoracic Surgery, Zhongshan Hospital, Shanghai Medical College, Fudan University, Shanghai, China; 3 Department of Nephrology, Hangzhou Hospital of TCM, Hangzhou, China; 4 Department of Radiology, Zhongshan Hospital, Shanghai Medical College, Fudan University, Shanghai, China; 5 Department of Physiology, Medical College of Wisconsin, Milwaukee, Wisconsin, United States of America; National Cancer Institute, United States of America

## Abstract

Mesenchymal stem cell (MSC) administration is known to enhance the recovery of the kidney following injury. Here we tested the potential of hypoxic-preconditioned-MSC transplantation to enhance the efficacy of cell therapy on acute kidney injury (AKI) by improving MSC migration to the injured kidney. Cobalt was used as hypoxia mimetic preconditioning (HMP). MSC were subjected to HMP through 24 h culture in 200 µmol/L cobalt. Compared to normoxia cultured MSC (NP-MSC), HMP significantly increased the expression of HIF-1α and CXCR4 in MSC and enhanced the migration of MSC *in vitro*. This effect was lost when MSC were treated with siRNA targeting HIF-1α or CXCR4 antagonist. SPIO labeled MSC were administered to rats with I/R injury followed immediately by magnetic resonance imaging. Imaging clearly showed that HMP-MSC exhibited greater migration and a longer retention time in the ischemic kidney than NP-MSC. Histological evaluation showed more HMP-MSC in the glomerular capillaries of ischemic kidneys than in the kidneys receiving NP-MSC. Occasional tubules showed iron labeling in the HMP group, while no tubules had iron labeling in NP group, indicating the possibility of tubular transdifferentiation after HMP. These results were also confirmed by fluorescence microscopy study using CM-DiI labeling. The increased recruitment of HMP-MSC was associated with reduced kidney injury and enhanced functional recovery. This effect was also related to the increased paracrine action by HMP-MSC. Thus we suggest that by enhancing MSC migration and prolonging kidney retention, hypoxic preconditioning of MSC may be a useful approach for developing AKI cell therapy.

## Introduction

Acute kidney injury (AKI) is defined by an abrupt and sustained impairment of renal function that can be initiated by various insults [1]. In China AKI affects 3% to 4% of the hospitalized patients, and approximately 13% of patients admitted to intensive care [2]. Ischemia-reperfusion (I/R) injury is one of the most common causes of AKI and is characterized by tubular necrosis and apoptosis. Clinical management of AKI, such as implementation of modern dialysis techniques, has improved significantly over the last years [3], but a specific therapy to improve renal function after AKI has not yet been developed. Complications arise from the inability of the kidney to repair tubular lesions through regeneration of functional tubular epithelial cells. Additionally, failure to repair this damage gives rise to tubulointerstitial fibrosis and increases the susceptibility to chronic kidney disease (CKD) [4], [5]. It is for these reasons that there is a critical need to develop specific interventions that promote renal tubular epithelial cell repair as a treatment of AKI.

While pharmacologic interventions often target only a single aspect of the highly complex pathophysiology following AKI, cell-based therapies may have the advantage of acting through multiple mechanisms to promote tubular epithelial cell repair [5]. Compared with conventional stem cells, MSC provide many advantages because of their ease of harvest, stable genetic background, lower risk of tumor formation, no immunogenicity and decreased ethical concerns [6], [7]. In the last few years, several studies have shown that MSC administration enhances structural and functional recovery of injured kidneys through mechanisms that include: 1) engraftment and differentiation of MSC into the host tissue or organ, 2) therapeutic fusion with existing host cells, 3) release of paracrine and/or endocrine signals from MSC that influence resident tubular cells and 4) stimulation of endogenous mechanisms that regenerate local resident stem cells [8]–[13]. These mechanisms have been studied to various degrees in the kidney but the relative importance of each for MSC induced renal recovery remains unclear. Despite the mechanism of action, strategies that increase the number of MSC within the injured kidney would significantly improve the beneficial effects of cell therapy.

Several mediators and receptors are involved in the migration of cells to sites of injury. Interaction between the chemokine stromal-derived factor 1 (SDF-1), and its receptor CXCR4 is of pivotal importance in this process [14]–[17]. CXCR4 is normally expressed in bone marrow (BM) stem cells and is up-regulated by hypoxia [18]–[21]. The high level of CXCR4 expression in BM cells is thought to result, at least partially, from the relatively low oxygen tension in BM [20], [22]. Therefore, increases in expression of CXCR4 on the surface of stem cells by hypoxic preconditioning may enhance the benefit of cell therapy for the treatment of renal injury similar to those results achieved in a myocardial infarction model in mice [23].

Based on this background, the present study tested the hypothesis that hypoxic preconditioning with cobalt would enhance the migration ability of MSC *in vitro* and would enhance the efficacy of cell therapy on AKI, in an I/R rat model through stimulation of MSC migration to the injured kidney.

## Results

### Characteristics of MSC

**Figure 1:**
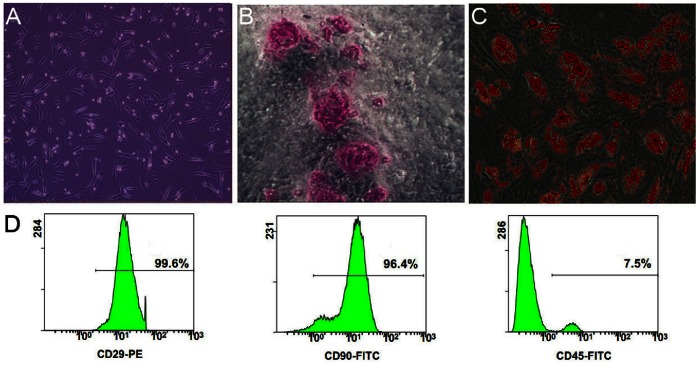
MSC Characterization. MSC had the ability to differentiate into osteocytes and adipocytes (A: MSC in culture; B: Fat cells: Sudan red; C: Bone cells: von Kossa stain), and expressed the surface markers CD29 and CD90, but not CD45 (D: flow cytometric analysis).

MSC were generated using standard procedures and grown for at least three passages in culture. The purified MSC continued to display a uniform fibroblast-like appearance throughout the culturing process (Figure 1A). Flow cytometric evaluation showed that the cell population was comprised of 99.6% CD29+ cells, 96.4% CD90+ cells and 7.5% CD45+ cells (Figure 1D), suggesting their mesenchymal rather than hematopoietic origin. Each batch of MSC was further characterized by confirming their specific ability to undergo osteogenic and adipogenic differentiation (Figure 1B and C).

### Hypoxic Mimetic Preconditioning Augmented the Expressions of HIF-1α and CXCR4 on MSC

Cobalt chloride (CoCl_2_) was used to mimic hypoxic preconditioning (HMP). We defined conditions for HMP as a culture concentration of 200 µmol/L CoCl_2_ for 24 hours (see Materials and Methods). After CoCl_2_ treatment for 24 h, neither the morphology (data not shown) nor the natural cell surface characteristics of MSC [CD29 (99.5%), CD90 (96.1%) and CD45 (7.7%)] were altered.

**Figure 2:**
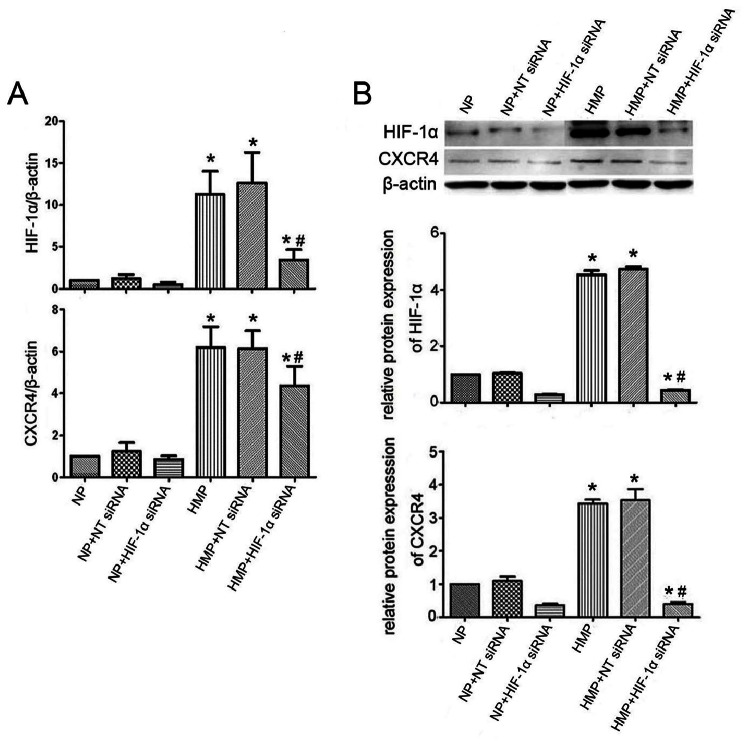
Hypoxia augmented the expressions of HIF-1α and CXCR4 on MSC. (A) Real-time RT-PCR analysis. (B) Western blot analysis. Abbreviations: NT siRNA, nontargeting siRNA. Values are presented as means ± SEM (n = 3 per group). *P<0.05, vs NP; #*P*<0.05, vs HMP.

As shown in Figure 2A, compared with normoxia preconditioning (NP), pretreatment with HMP significantly increased the mRNA and protein expression of HIF-1α and CXCR4 in MSC, and transfection of MSC with HIF-1α siRNA impaired HMP-induced HIF-1α and CXCR4 expressions. Western blot analysis had the similar results (Figure 2B).

### Hypoxic Mimetic Preconditioning Enhanced MSC Migration in vitro

**Figure 3:**
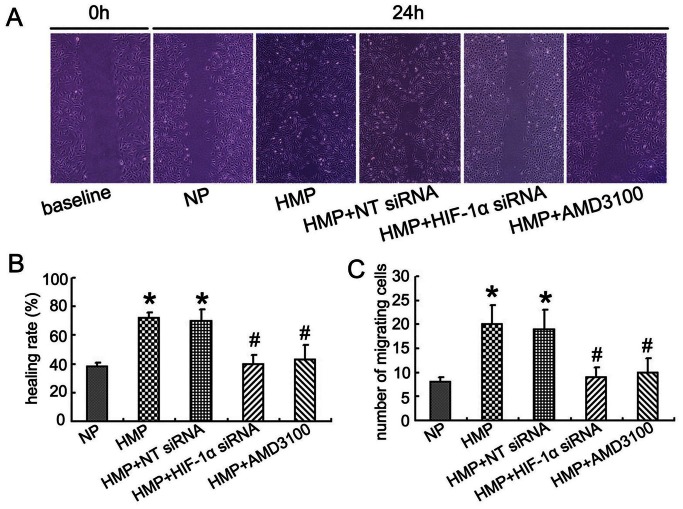
Effect of hypoxia mimetic pretreatment on MSC migration. (A) Scratch-wounding healing assay (magnification ×200). (B) Healing rate of scratch assay. (C) The number of migrating cells in Transwell assay. HMP pretreatment significantly increased the healing rate and in vitro migration ability of MSC, while down-regulation of HIF-1α by siRNA or suppression of CXCR4 by AMD3100 reversed this effect. *P<0.05, vs NP; #*P*<0.05, vs HMP.

We examined whether migration of MSC, an essential early step in the process of paracrine and differentiation of MSC in the injured organ, is enhanced under HMP. Scratch-wounding healing assay revealed that HMP significantly increased the mean healing rate of MSC from 38.2% to 71.4% (*P*<0.05). By contrast, either siRNA mediated knockdown of HIF-1α or AMD3100 incubation inhibited the effects of HMP on wound healing ability (Figure 3A and B). Similarly more HMP-MSC migrated through the pores of membrane at the bottom of the upper wells to the opposite side of the membrane, and this effect was removed by siRNA HIF-1α or AMD3100 incubation (Figure 3C).

### In vitro Cell Labeling

We used either Fe_2_O_3_-PLL nanoparticles (SPIO) or fluorescent dye CM-DiI to label MSC. Prussian blue staining of cultured SPIO-MSC after 24 hours of incubation in the suspension of SPIO particles (30 µg/ml iron) revealed an effective intra-cytoplasmatic uptake of iron oxide nanoparticles (97.5±3.2% of cells) indicated by blue-stained vesicles within the cells (Figure S1A). Fluorescence of CM-DiI-labeled MSC also showed a strong red signal at 600 nm (Figure S1B, 98.1±3.9% of cells). Trypan blue staining showed that either SPIO loading or CM-DiI labeling had no adverse effect on cell viability when compared to non-labeled control MSC (viability: SPIO-MSC 94.7%±6.5%, CM-DiI-MSC 96.1%±5.9%, versus control 95.0%±6.9%, *P*>0.05).

### In vivo MR Imaging of the Labeled MSC

**Figure 4:**
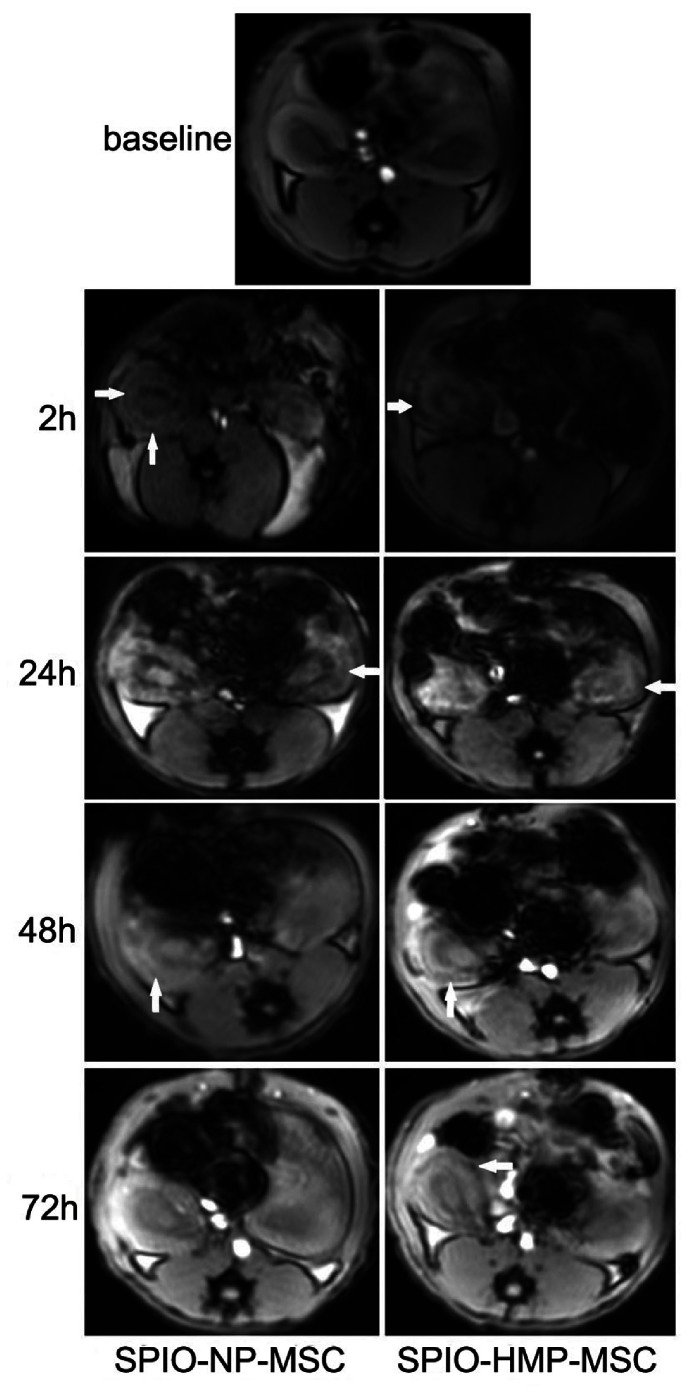
T2*-weighted MR images before (baseline), 2 hours, 24 hours, 48 hours and 72 hours after the injection of labeled MSC. The ischemic kidneys showed distinct decreased SI (arrows) in cortex after injection indicating localization of iron-labeled MSC. The SI reduction was more significant in HP-MSC group than in NP-MSC group at the corresponding time point. In the NP- MSC treatment group SI decrease was attenuated after 2 hours and returned to normal at 72 hours, while the SI reduction in the HMP-MSC treatment group persisted 72 hours after injection.

**Figure 5:**
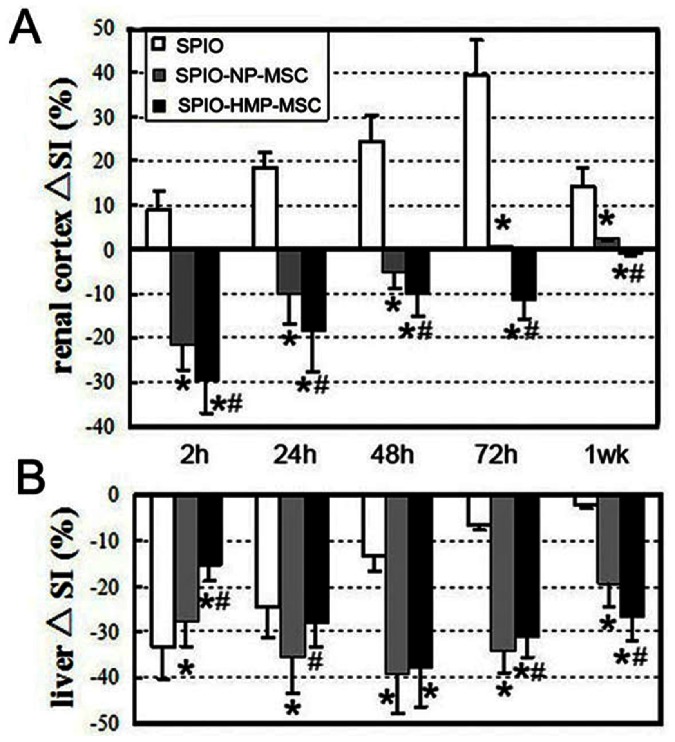
SI changes (ΔSI) of renal cortex and liver after the injection of SPIO or SPIO labeled MSC. ΔSI (%) = (SI[post]−SI[baseline])/SI[baseline]×100. (A) SI of the renal cortex of HMP-MSC treated animals showed a significant decrease after administration of labeled MSC and a prolonged signal reduction up to week 1. In contrast, the ΔSI of the renal cortex in NP-MSC group was lower than that of the HMP-MSC group, and the signal reduction was continued only to 72 h. Non-treated (SPIO) animals demonstrate an SI increase after AKI, which is caused by renal edema. (B) In the liver, the rapid accumulation of SPIO particles in Kupffer cells lead to a maximum of SI decrease immediately after particle injection (2 h). After application of SPIO-MSC the maximum SI reduction was observed at 48 h, demonstrating the delayed accumulation of stem cells in the liver tissue. The SI reduction in liver was lower in HMP-MSC group than in NP-MSC group prior to 48 h, indicating a delayed accumulation of stem cells in kidney after hypoxic preconditioning. *P<0.05, vs SPIO; #*P*<0.05, vs SPIO-NP-MSC.

The accumulation of SPIO labeled cells in a tissue induces a local magnetic susceptibility gradient resulting in a decrease in MR signal intensity (SI) in that tissue. Before ischemia (Figure 4 baseline), a lower SI was observed around the renal pelvis on T2*-weighted MR images. After reperfusion, the renal cortex even showed an SI increase (data not shown), but after injection of the labeled cells, SI loss in the outer zone of the renal cortex, which was produced by susceptibility effects of SPIO, was demonstrated on T2*-weighted MR images 2 h after transplantation in comparison to baseline. Additionally, this SI loss was greater in the HMP-MSC group than in NP-MSC group (Figure 4 and Figure 5A). The SI decrease in the renal cortex was gradually normalized over 72 hours in NP-MSC group indicating a loss of labeled cells. Until 1 week later did the SI decrease lose in HMP-MSC group, suggesting a greater cell retention in this group. At no time point could an SI decline be observed in the renal cortices of SPIO control animals. The SI of renal medulla did not change in any of the groups at any point in the experiment (data not shown).

The liver demonstrated a reduction of SI in all three groups, with a rapid loss of SI in the SPIO control animal immediately (2 h) after SPIO application and a delayed SI decrease in both MSC groups. The SI decrease reached a maximum at 48 h after MSC injection with the SI greater in NP-MSC group than in HMP-MSC group at 2 h, 24h, 72 h and 1 week after transplantation (Figure 5B).

### Correlation between Histology and MRI

**Figure 6:**
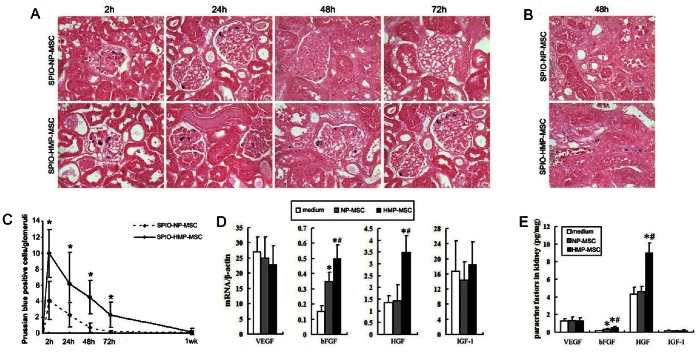
HMP enhanced the retaining and paracrine actions of MSC in the I/R kidney. (A) Prussian blue staining of renal cortex after AKI and application of labeled MSC showed a predominant presence of cells in the glomeruli corresponding to the regions of signal loss in MRI. The labeled MSC was quantified by counting the number of Prussian blue staining-positive cells per glomeruli in 20 non-sequential sections under ×200 magnification (C).The number of MSC migrated to the kidney cortex was higher and the retention time was longer in HMP-MSC group than in NP-MSC group. *P<0.01, vs SPIO-NP-MSC. (B) Prussian blue staining of tubulointerstitium 48 h after MSC injection. Few iron-labeled NP-MSC were found in the tubulointerstitial tissue. More iron-labeled MSC were found in the tubulointerstitial in HMP-MSC group than in NP-MSC group. (Magnification ×400) The mRNA levels and protein levels of paracrine factors in the kidney were determined by (D) real-time RT-PCR and (E) ELISA separately. The expressions of bFGF and HGF were higher in HMP-MSC group than NP-MSC group. *P<0.05, vs medium; #*P*<0.05, vs NP-MSC.

As shown in Figure 6A and C, positive Prussian blue staining, the characteristic phenotype of the iron-labeled MSC, was detected in the glomerular capillaries in the outer zone of the renal cortex, as early as 2 h after transplantation in both MSC groups, corresponding to the SI reduction in MR images. The degree of positive staining per glomeruli was more in HMP-MSC group than in NP-MSC group. Few iron-labeled MSC were found in the tubulointerstitial tissue in either group. By 48 h, occasional cells were detected in the NP-MSC treated kidney; the number of MSC in HMP-MSC group also declined but was still moderate in glomerulus, and was even higher in the tubulointerstitial compartment (Figure 6B). At 72 h after cell infusion, no cells could be detected in the kidney in the NP-MSC group; however, in the HMP-MSC group cells were still detectable in glomeruli and the tubulointerstitium until 1 week post-injection.

**Figure 7:**
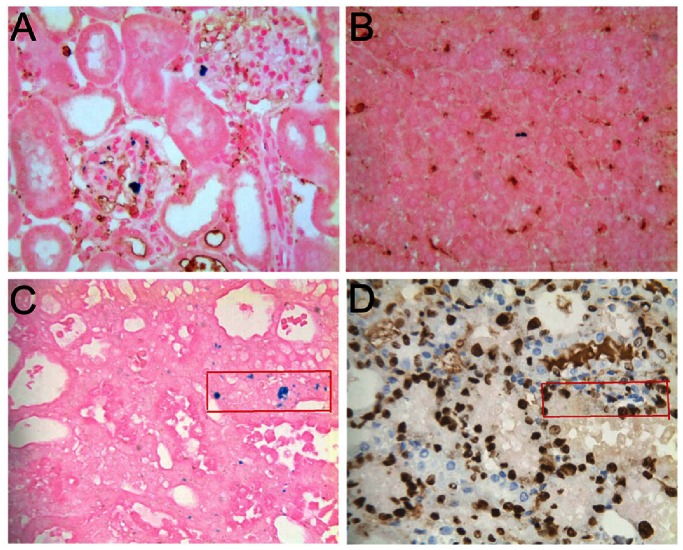
Characteristics of labeled MSC in kidney and liver. Double staining for Prussian blue and CD68 of kidney (A) and liver (B) demonstrates that SPIO-labeled cells are not macrophages. The consecutive sections staining for iron (C) and Ki-67 (D) of the kidney 48 h after HMP-MSC injection indicated that SPIO-labeled cells in tubulointerstitium had a proliferative capacity. (Magnification ×400).

Negative CD68 staining of Prussian blue positive cells in kidney and liver sections of the SPIO-labeled MSC group confirmed that these cells were SPIO labeled MSC and not macrophages (Figure 7A and B). Furthermore, absent SI reduction in MRI corresponded with the lack of SPIO accumulating cells in the kidneys after free SPIO particle injection. This excluded the presence of SPIO phagocytosing cells such infiltrating macrophages. Besides, the consecutive sections staining for iron and Ki-67 of the kidney 48 h after HMP-MSC injection indicated that the Prussian blue positive cells in tubulointerstitium had a proliferative capacity (Figure 7C and D).

### Further Detection of Administered MSC by Fluorescence Microscopy

**Figure 8:**
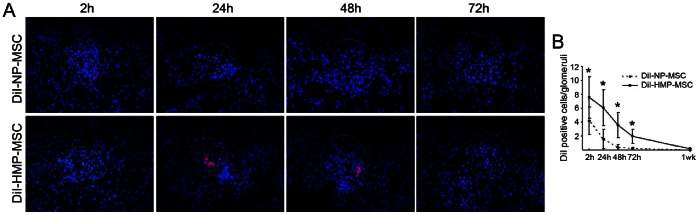
MSC detection in kidney by fluorescence microscopy. (A)MSC was labeled with CM-DiI and appeared red under fluorescent microscopy (nuclei stained blue with DAPI). The labeled cells were mainly detected in glomeruli (dashed circles) after injection. Magnification ×400. (B) CM-DiI labeled MSC was quantified by counting the number of red staining cells per glomeruli in 20 non-sequential sections under ×200 magnification. The number of MSC migrated to the kidney cortex was higher and the retention time was longer in HMP-MSC group than in NP-MSC group. *P<0.01, vs DiI-NP-MSC.

To further evaluate the renal delivery of intra-arterially infused MSC and to verify MRI results, additional I/R rats were killed before ischemia, and 2 h, 24 h, 48 h, 72 h and 1 week after CM-DiI-labeled NP-MSC or HMP-MSC administration, and the kidneys were examined for the presence of MSC by fluorescence microscopy (n = 5 per time point). As shown in Figure 8, CM-DiI-labeled MSC was appeared inside glomerular capillaries as early as 2 h after infusion, and disappeared 24 h later after administration in NP group, but in HMP group, more cells were located in the glomerular capillaries and the retention time extended to 72 h after transplantation, which was consistent with the result of MRI and Prussian blue staining.

### Therapeutic Potential of Hypoxic Mimetic Preconditioned MSC

**Figure 9:**
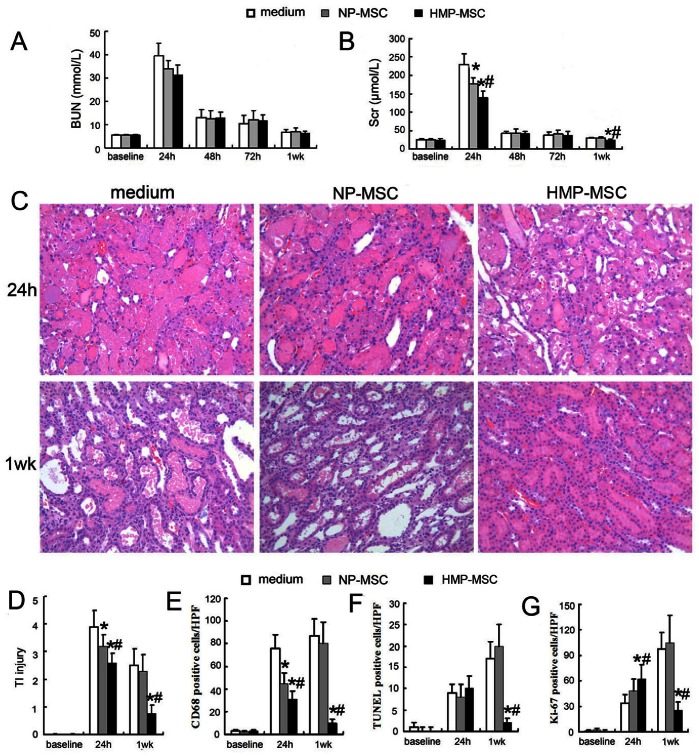
HMP-MSC injection protected the kidney from I/R injury better than NP-MSC administration. Renal function was evaluated by determining (A) Scr and (B) BUN levels in the week following I/R AKI. Administration of HMP-MSC had a better maintained renal function than NP-MSC and vehicle treatment at 24 h and 1 wk following transplantation. (C) Renal morphological changes after I/R injury (H&E-stainning, magnification ×200). (D) Tubulointerstitial (TI) injury score in the cortex and outer medullar region. HMP-MSC administration significantly lowered TI injury score at 24 h and 1 wk after reperfusion. (E) HMP-MSC rats showed lower numbers of CD68 positive macrophages than medium and NP-MSC groups at 24 h and 1 wk after reperfusion. (F) TUNEL positive apoptotic cells in the three groups were not significantly different at 24 h after injection, but it was significantly lower in HMP-MSC group than in medium and NP-MSC groups 1 week after injection. (G) The number of Ki-67 positive proliferating cells was higher in HMP-MSC group at 24 h after injection with most of these cells localized in tubules (Figure 10). Conversely, the number of Ki-67 positive cells was lower in HMP-MSC group 1 week after injection with most of the remaining located in interstitium (Figure 10). *P<0.05, vs medium; #*P*<0.05, vs NP-MSC.

After observing the dramatic HMP-induced increase in MSC migration to the cortex of the ischemic kidney, we investigated whether HMP enhanced the therapeutic benefit of MSC. As shown in Figure 9A and B, blood urea nitrogen (BUN) and serum creatinine (Scr) levels of control rats peaked at 24 h post I/R injury and recovered thereafter with lower BUN and Scr levels. Animals that had received HMP-MSC had a much lower Scr levels than control animals at 24 hours. Interestingly, administration of NP-MSC also resulted in lower 24 h Scr level; however this effect was weaker than in the HMP rats. At week 1, the Scr levels between NP-MSC treated rats and control rats did not reach a statistically significant difference, however HMP-MSC administration improved renal function compared to other groups.

**Figure 10:**
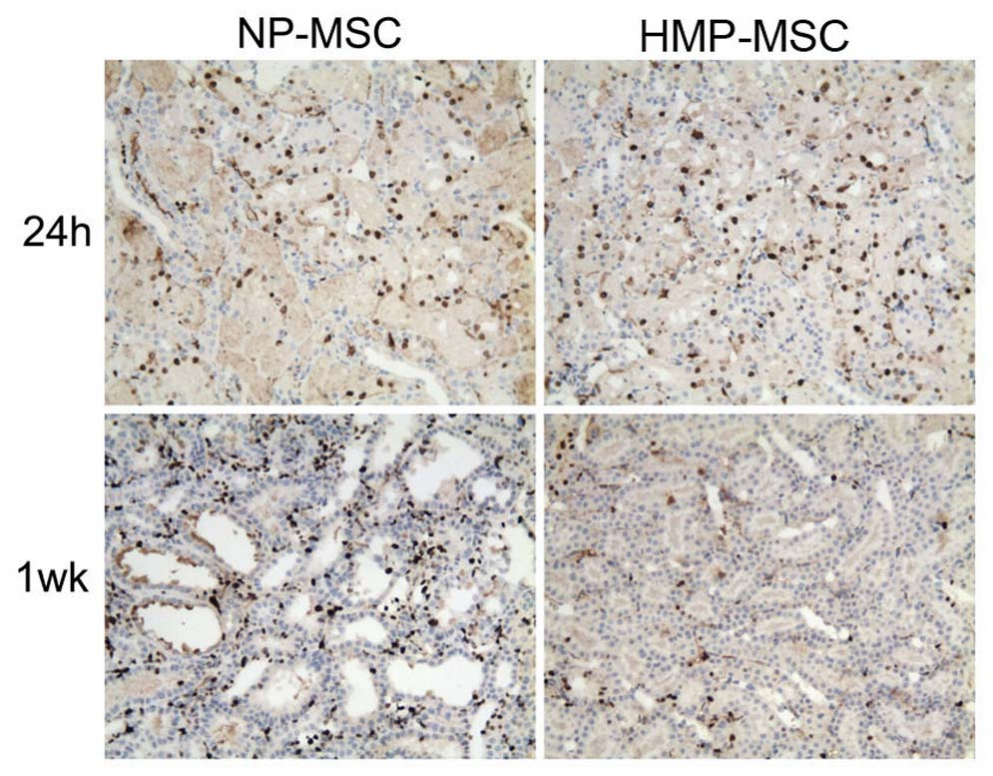
Tubulointerstitial cell proliferation after MSC injection. Cell proliferation was analyzed immunohistochemically by Ki-67 staining. At 24 h after MSC injection the number of Ki-67 positive cells was higher in HMP-MSC group than in NP-MSC group, with most of these cells localized in tubules. Conversely, the number of Ki-67 positive cells was lower in HMP-MSC group than in NP-MSC group 1 week after injection, and most of the positive cells were located in interstitium. (Magnification ×200).

Rats were sacrificed at 24 h and 1 week post IR injury for scoring of renal injury, macrophage infiltration (CD68 staining), tubular epithelial cell proliferation (Ki-67 staining), and cell apoptosis (TUNEL staining) (Figure 9C–G). At 24 h, acute tubular necrosis, loss of brush border, tubular dilatation and cast formation in the cortex and outer medullar region were minimized in kidneys from rats administered HMP-MSC when compared to rats treated with NP-MSC. Kidneys treated with HMP-MSC were also associated with less CD68 positive macrophage infiltration and more Ki-67 positive tubular epithelial cell proliferation (Figure 10). At week 1, renal tissue injury was still evident both in control and NP-MSC treated groups. This injury included tubular dilation, tubulointerstitial edema, expansion of the interstitial compartment, a tendency for interstitial fibrosis (more Ki-67 positive cells in interstitial compartment, Figure 10), macrophage infiltration and cell apoptosis. This suggested that administration of NP-MSC transplantation cannot reverse the morphologic injury caused by ischemia despite significantly lower Scr levels at 24 h. In contrast, animals receiving HMP-MSC exhibited normal renal morphology upon histological examination.

### Paracrine Factors in MSC-infused Kidneys

Given the importance of paracrine and/or endocrine effects of MSC in renal protection, we determined the expressions of vascular endothelial growth factor (VEGF), basic fibroblast growth factor (bFGF), hepatocyte growth factor (HGF) and insulinlike growth factor 1 (IGF-1), four typical cytokines and growth factors, in kidneys 24 h after MSC injection. As shown in Figure 6D, the mRNA level of bFGF was higher in NP-MSC group than in control group, and no significant differences were seen in VEGF, HGF and IGF-1 between the two groups. However, HMP-MSC group had even higher levels of bFGF and HGF than NP-MSC group. The protein expressions of the above factors were confirmed by ELISA (Figure 6E).

## Discussion

The current study demonstrated that HMP by cobalt increased MSC migration *in vitro* that depended on the activation of HIF-1α and the up-regulation of its target gene CXCR4. In addition MR imaging and histological examination of tissue revealed that systemic administration of HMP-MSC migrated to I/R kidneys more efficiently and were retained for a longer time than NP-MSC. Finally, HMP-MSC also reduced kidney damage and promoted the recovery of kidney from I/R injury to a greater degree than either NP-MSC or vehicle alone, and this beneficial effects of HMP-MSC are also mediated by a better paracrine actions.

The issue of the optimum oxygen tension in which to culture MSC has been under investigation for many years. Physiological oxygen tension varies from as high as 12% in the blood to as low as 1–2% in bone marrow. Once localized to the ischemic tissue, MSC encounter more severe hypoxic conditions, ranging from 0.4% to 2.3% O_2_, which often results in apoptosis [24]. In any case, the oxygen tension is considerably lower *in vivo* than the atmospheric oxygen tension (21%) of standard cell culture, so hypoxic preconditioning *in vitro* is currently employed as a strategy to prepare MSC for increased survival and engraftment in the ischemic tissue. In the present study we used cobalt to mimic hypoxia *in vitro*. Cobalt has been suggested to induce the “hypoxia-mimetic” effect by substituting for iron on an oxygen sensing heme protein as well as by depleting intracellular ascorbate [25], [26]. Both iron and ascorbate are important substrates of prolyl hydroxylases, the key enzymes involved in HIF-α degradation. Cobalt has now been widely used to activate HIF-α *in vitro* and *in vivo*, and cobalt and hypoxia effects on the HIF-α target gene may be quite similar [27]–[31]. The effect of hypoxia on stem cells can vary widely with differences in the oxygen concentration and duration of exposure. In the current study we induced a hypoxia-mimetic response in cultured MSC with an optimized cobalt concentration and duration. We observed that when MSC were cultured in 200 µmol/L cobalt for 24 h, cell proliferation was not significantly suppressed, and cell apoptosis was not significantly increased, when compared with MSC pretreated in normoxia. Higher concentrations of cobalt and prolonged conditioning induced obvious cell death. Similar results were observed in Chacko’s study where hypoxia treatment of MSC (0.5% O_2_) for 24–48 h was not a sufficient stimulus to negatively affect the cells’ proliferation status, however a longer exposure (>72 h) could induce apoptosis [32]. In fact, after overcoming a lag phase in proliferation upon the initiation of hypoxic culture conditions, hypoxic-MSC have been shown to proliferate faster than MSC cultured at 21% O_2_ [33].

Consistent with other reports [16], [34]–[36], our *in vitro* study confirms that the up-regulation of CXCR4 after HMP acts as a motogenic factor for MSC and increased their migration potential. Receptor CXCR4 and its ligand SDF-1 are of pivotal importance in migration of MSC to sites of injury. Expression of CXCR4 declines following a few passages in culture which likely decreases the homing and engraftment potentials of cultured MSC in most cell therapy studies where cell expansion is unavoidable [37], [38]. To overcome this limitation the overexpression of CXCR4 on the surface of stem cells has been examined by investigators in an attempt to better understand its regulation [34]–[36]. CXCR4 is one of the target genes of HIF-1α and it has been well documented that hypoxia-induced expression of CXCR4 in various tumor cells is regulated by the activation of HIF-1α, thereby promoting the aggressiveness of tumor [39]–[41]. Similarly, short-term exposure of stem cells to hypoxia also up-regulates their expression of CXCR4 by activating of HIF-1α, leading to migration of stem cell [23], [34], [35]. We found that HMP significantly induced the expression of HIF-1α and CXCR4, and improved the migration of MSC *in vitro* in a HIF-1α dependent manner.

Labeling of cells with SPIO has been shown to be a nontoxic and long-lasting method for *in vivo* tracking of stem cells using MR imaging thus allowing the location of systemically administered cells to be visualized over time. A significant advantage of SPIO labeling is the preservation of the ability of labeled MSC to differentiate into adipocytes and osteocytes. Several previous studies have reported encouraging results in tracking of SPIO-labeled stem cells to determine their biodistribution and migration in different organs, including kidney [42]–[44]. In the present study, we also used MR imaging and Prussian blue staining to detect the migration of SPIO labeled MSC. Besides, another traditional molecular probe CM-DiI was also applied to label and track MSC. We confirmed that therapy with NP-MSC afforded renal protection in rats with I/R AKI, which has been reported by others [13], [44]. Although this effect was a little weaker than reported, we presumed that a more severe I/R rat model and a relatively smaller number of MSC may be the reasons. Rats treated with NP-MSC lacked iron-staining in the tubules and vascular endothelial cells, with the majority of labeled MSC localizing to glomeruli. Additionally, because of lower indices of renal damage (Scr) at 24 h, we hypothesize that transdifferentiation of administered NP-MSC into renal cells is not the primary renal protective mechanism, because this process takes more than days [44]. Interestingly, we found that HMP-MSC treatment afforded greater protection to the kidney than NP-MSC in I/R rats. In agreement with observations from our *in vitro* study, HMP enhanced the migration capacity of MSC toward the injured kidney. Furthermore, in addition to the majority of labeled HMP-MSC locating in the glomeruli, a small number of injected cells also appeared in the tubulointerstitium of the cortex and outer medullary regions 48 h after transplantation. These cells were negative for the macrophage marker CD68 and cell proliferation marker Ki-67, confirming that HMP-MSC had implanted in the tubulointerstitium and had a proliferative capacity. Also HMP-MSC administration up-regulated the expressions of angiogenic factor bFGF and antiapoptotic and mitogenic factor HGF in kidneys than NP-MSC injection, suggesting that HMP-MSC had a better paracrine actions than NP-MSC. Whether the administered HMP-MSC that migrated to the tubulointerstitium participate in renal repair by transdifferentiation into tubular epithelial cells and/or through a local paracrine effect remains to be elucidated.

The present study must be interpreted within the context of certain limitations. Since cobalt is taken up by MSC, it cannot be ruled out that retained cobalt is released after MSC are recruited to the injured kidney. Cobalt now has been known to be beneficial in AKI and CKD by stimulating the production of HIF target genes such as erythropoietin, vascular endothelial growth factor and heme oxygenase-1 [29], [31], [45], [46]. Because the dose of the probably released cobalt in our study (less than 0.01 mg/animal) is far less than the effective dose reported, we could exclude the influence of cobalt on the result that HMP-MSC transplantation enhances the therapy for the treatment of AKI. Furthermore, cobalt is toxic to the organic body. Although the dose of the probably released cobalt in our study is also much lower than toxic dose [47], and we failed to observe toxic effects in terms of gross hematuria and glomerular capillaries filled with red blood cells [47], the benefits of hypoxic preconditioning with cobalt of MSC given in clinical practice to AKI is still limited by its toxicity. The future availability of environmental hypoxia preconditioning might open new therapeutic avenues.

In conclusion, our study suggests that HMP of MSC before implantation could enhance migration ability by activating HIF-1α and up-regulation of CXCR4, resulting in increased migration of transplanted MSC to the injured kidney and renal protective factors paracrined into the injured kidney and promoting improved morphology and function following AKI. Because hypoxia preconditioning can positively alter cell behavior without the use of additional growth factors, viruses, or other non-clinically relevant agents, it offers a promising approach to the clinic treatment of AKI.

## Materials and Methods

### Ethics Statement

Male Sprague-Dawley rats were provided by the Animal Center Shanghai Medical College, Fudan University (Shanghai, China). This study was carried out in strict accordance with the Guidelines on the Care and Use of Laboratory Animals issued by the Chinese Council on Animal Research and the Guidelines of Animal Care. The protocol was approved by the Committee on the Ethics of Animal Experiments of Fudan University. All efforts were made to minimize animals’ suffering and to reduce the number of animals used.

### MSC Isolation, Culture and Identification

MSC were generated from the bone marrow of adult male Sprague-Dawley rats aged 6 weeks weighing 120–150 g by standard procedures. Briefly, the bone marrow aspirate were obtained by flushing the rat femurs and tibias with FBS free LG-DMEM medium (Gibco, Invitrogen, CA, USA), and was then centrifuged and re-suspended in LG-DMEM medium with 10%FBS and incubated at 37°C in a humidified chamber with 5% CO_2_. The culture media was completely replaced every 3 days and non-adherent cells were discarded. MSC were recognized by their ability to proliferate in culture with an attached well-spread morphology. Once cells were more than 80% confluent, adherent cells were detached and re-plated 1∶3 by flask.

MSC were defined by the expression of specific membrane markers (CD29+, CD90+, CD45−) using cytofluorimetric analysis with a flow cytometer (Beckman Coulter, CA, USA) [48]. The primary mouse anti-rat monoclonal antibodies used for identification were anti-CD29 (clone HMβ1-1), anti-CD90 (clone OX-7), and anti-CD45 (clone OX-1) all from Biolegend, CA, USA. The multipotency of MSC was confirmed by induction of osteogenic and adipogenic differentiation using specific differentiation media as previously described [49].

### Hypoxic Mimetic Treatment of MSC

Cobalt chloride (CoCl_2_) was used as hypoxic mimetic preconditioning (HMP). Passage 3 (P_3_) MSC were incubated with CoCl_2_ at different concentrations (0–500 µmol/L) and durations (6–72 h) to determine the optimum treatment conditions for subsequent experiments. MSC proliferation was measured by MTT analysis and cell apoptosis (Annexin V) was measured by flow cytometry (see below). These results indicated that exposure to concentrations higher than 200 µmol/L CoCl_2_ for more than 24 hours had lower viability (*P*<0.05) (see Figure S2), so in all further experiments we defined conditions for HMP as a culture concentration of 200 µmol/L for 24 hours, followed by a two hour incubation in CoCl_2_ free medium.

### Measurement of Cell Proliferation by MTT

Passage 3 (P_3_) MSC were incubated into 96-well plate with 1×10^6^ cells in each well under normoxia. 24 h later, 100 µL CoCl_2_ solution at concentrations of 0, 50, 100, 150, 200, 300, 400 or 500 µmol/L were added into each well. Cell viability at 6 h, 12 h, 24 h, 48 h and 72 h was then examined using MTT method. OD550 nm values at each time point were detected in six duplicate wells and their averages were used to plot growth curve and calculate the growth inhibition rate of each treatment using the following formula: Growth inhibition rate = (OD550 nm of the control group - OD550 nm of the treatment group)/OD550 nm of the control group×100%.

### Measurement of Cell Apoptosis by Flow Cytometry

After CoCl_2_ conditioning, MSC were collected, washed with PBS, resuspended in PBS at 1×10^6^/mL, and stained with Annexin V and propidium iodide solution (PI) for 15 min in the dark. Apoptotic cells were then analyzed by flow cytometry and apoptotic index (AI) was calculated using AI = apoptotic cells/total cells ×100%.

### In vitro Scratch-wound Healing Assay

P_3_ MSC density was adjusted to 2×10^5^ cells/L and seeded into six-well plates. After formation of a monolayer, cells were scratched with a 10 µL pipette tip and cultured in normoxia. 24 h after scratching, cells in each well were photographed under microscope. The distance between the two edges of the scratched cells in four fields was measured and the average distance was used to calculate the healing rate using the following formula: Healing rate = (the distance before healing - the distance after healing)/the distance before healing ×100%.

### Transwell in vitro Migration Assay

P_3_ MSC were resuspended in FBS free LG-DMEM media and adjusted to a density of 1×10^6^/mL. 100 µL of cell suspension was added into the upper chamber of the transwell and 600 µL cell suspension containing 10% FBS was added into the lower chamber of the transwell. The transwell was then cultured at 37°C in an incubator supplemented with 5% CO_2_. 24 h later, cells on the upper surface of polycarbonate membrane of the transwell were removed with a cotton swab and the cells that migrated onto the lower surface of the membrane were fixed with 4% paraformaldehyde for 30 min, washed twice with PBS for 5 min and stained with crystal violet for 15 min. After washing with PBS, the membrane was air dried and cell was counted under microscope at 400× magnification. The number of migrated cells was expressed as the average of five randomly selected fields.

### Small Interference RNA Transfection

Synthetic siRNA oligonucleotide specific for HIF-1α (NM_024359) (5′ to 3′: UUUAUCAAGAUGGGAGCUCTT) and a nontargeting siRNA were obtained from GenePharma (Shanghai, China). For transfection, MSC were incubated with siRNA complexes in FBS free media for 24 hours by using a Silence siRNA Transfection kit LipofectamineTM 2000 (Invitrogen, CA, USA) and then the medium was changed.

### AMD3100 Culture

P_3_ MSC were incubated with CXCR4 antagonist AMD3100 (5 ug/ml, Sigma, USA) at 37°C in an incubator supplemented with 5% CO_2_ for 6 h before CoCl_2_ or normoxia treatment.

### Western Blot

Protein abundance level of HIF-1α was examined by Western blot using nuclear proteins, and CXCR4 using total proteins. Nuclear proteins and total proteins were extracted from MSC according to the manufacturer’s instructions (Nuclear Extract kit, Active Motif, CA, USA), and protein concentrations were measured using a BCA protein assay kit (Kaiji Biotechnology, Nanjin, China). Protein samples (30 µg/Lane) were separated on 10% SDS–PAGE gels and then transferred to polyvinylidine difluoride (PVDF) membranes. Specific band was detected with anti-HIF-1α antibody (1/250, Abcam, Cambridge, UK), or anti-CXCR4 antibody (1/500, Abcam, Cambridge UK), followed by incubation with horseradish peroxidase-conjugated anti-rabbit secondary antibodies.

### Cell Labeling and Identification of the Labeled Cells

Prior to injection, MSC were labeled either with SPIO or the fluorescent dye CM-DiI (Molecular Probes, Invitrogen, USA). SPIO was a gift from Pro. N Gu (Laboratory of Molecular and Biomolecular Electronics, Southeast University, Nanjing, China) [50]. The P_3_ MSC was incubated with 30 mg/ml SPIO continuously for 24 h at 37°C in a 95% air per 5% CO_2_ incubator as recommended. Prussian blue staining was used to estimate the efficiency of SPIO uptake and localization of the iron oxide particles within the cells. CM-DiI labeling was performed according to the manufacturer’s instruction and the labeling efficiency was estimated by fluorescence microscopy [51]. Trypan blue staining was used to determine the viability of MSC after labeling, as described previously [50].

### Animals, Induction of I/R AKI, and MSC Transplantation

Models of I/R AKI were performed with male Sprague-Dawley rats weighing 350–400 g. All the surgical procedures were carried out under general anesthesia (sodium pentobarbital, 40 mg/kg, i.p.) After a midabdominal laparatomy, kidneys were exposed and renal pedicles were clamped with atraumatic vascular clamps for 45 min. While clamps were applied, the left carotid artery was cannulated with a safety IV catheter (24 G) for intra-aortic cell delivery immediately after reflow. Control rats with AKI received vehicle (FBS free LG-DMEM) by the same route.

The animals were randomly assigned to one of three experimental groups as follows: group 1, AKI animals treated with 0.5 ml FBS free LG-DMEM (control); group 2, AKI animals treated with 1×10^6^ labeled normoxia preconditioned (NP) MSC in 0.5 ml FBS free LG-DMEM (NP-MSC); group 3, AKI animals treated with 1×10^6^ HMP-MSC in 0.5 ml FBS free LG-DMEM (HMP-MSC). Equal numbers of rats in each of the three groups were randomly euthanized at 24 h, 48 h, 72 h and 1 week after injection (n = 6 per time point). To study the in vivo bio-distribution of free SPIO particles in AKI rats, another 12 I/R rats were injected with 0.5 ml SPIO (diluted in FBS free LG-DMEM at 30 mg/ml) immediately after reperfusion and were euthanized at 24 h, 48 h, 72 h and 1 week (n = 3) after injection. MRI scans were performed three days before AKI (pre) and just before sacrifice at each time point.

Rats were terminally anesthetised using sodium pentobarbital at the indicated times. Blood samples were collected via cardiac puncture. Kidneys were cut longitudinally in halves to be snap frozen in liquid nitrogen, or fixed in paraformaldehyde and paraffin embedded, or embedded in OCT and cryosectioned.

### MR Imaging

We used MR imaging to study the *in vivo* migration of SPIO-labeled MSC. All MR imaging was performed with a 3-Tesla clinical unit (MAGN ETOM Verio, Siemens Healthcare). A special animal coil (Siemens Healthcare) with a diameter of 5 cm was used to scan the kidneys and liver of rats. T2* weighted gradient echo sequences (repetition time = 230 ms, echo time = 2.31, 11.90, 19.44, 26.98, 34.52, 41.47 ms; flip angle = 60°) were used to enhance the T2* effects of the SPIO particles. The following image parameters were used: field of view, 120×120 mm; matrix size, 192×192; section thickness, 3 mm; number of excitation, 5; total acquisition time, 2.1 minutes. Both kidneys were imaged in the axial plane.

MR images of the kidneys and liver of the recipients were obtained before ischemia (baseline), and 2 h, 24 h, 48 h, 72 h and 1 week after MSC transplantation, respectively. The edge of renal cortex was outlined and mean SI from four regions of interest in the liver and each kidney were evaluated by one of the authors (S Rao), an experienced radiologist, who was blinded to the transplanting procedure. SI changes (ΔSI) in each rat were calculated according to the following formula: ΔSI = (SI[baseline]- SI[post])/SI(baseline) ×100%. SI [baseline] and SI [post] were normalized compared to a region of interest in the back muscle.

### Assessment of Renal Function

BUN and Scr levels were determined using commercial kits (Urea FS, DiaSys Diagnostic System GmbH, Germany; CREA plus, Roche, USA) by an automatic analyzer (Hitachi 7180; Hitachi, Tokyo, Japan).

### Histology

Kidneys were fixed in 10% formalin and embedded in paraffin. Two-micrometer-thick sections were processed for hematoxylin and eosin staining. Abnormalities were graded using a semi-quantitative scale from 0 to 4, according to tubular cell necrosis, tubular dilation, cellular or proteinaceous casts, interstitial oedema and interstitial leukocyte infiltration. Higher scores represented more severe damage: 0, normal kidney; 1, <25% involvement of the cortex or outer medulla; 2, 25–50% involvement of the cortex or outer medulla; 3, 50–75% involvement of the cortex or outer medulla; and 4, >75% involvement of the cortex or outer medulla [42]. SPIO labeled MSC in kidneys and liver were identified with standard Prussian blue staining [50]. MSC migration to the kidney was quantified by counting the number of Prussian blue staining-positive cells per glomeruli in 20 non-sequential sections under ×200 magnification of cortex for each kidney.

### Immunohistochemistry

Immunoperoxidase staining was performed as described previously [42], with the following antibodies: macrophage infiltration was identified with monoclonal anti-CD68 (1/100; Chemicon, Temecula, USA) and cell proliferation was analyzed with monoclonal anti-Ki-67(1/100, Abcam, Cambridge, UK). Cell apoptosis was identified using an In Situ Cell Death Detection Kit (Roche, Mannheim, Germany) according to the manufacturer’s instructions. Scoring for CD68-positive cells, Ki-67-positive cells, or TUNEL-positive cells was carried out by counting the number of positive cells in 20 randomly chosen areas under ×200 magnification of the tubulointerstitium in the cortex or outer medulla for each kidney. To determine the SPIO accumulation by macrophages, sections were double-stained for Prussian blue and CD68 [43].

### Real-time RT-PCR

Total RNA was extracted from MSC using Trizol reagents (Invitrogen Life Technologies) according to manufacturer’s instructions. Reverse transcription was performed using the Takara RT-PCR kit. Real-time PCR was performed using the SYBR® Premix Ex Taq™ (Takara, Japan) and ABI PRISM 7900 HT Sequence Detection System according to standard protocols. To control for equal input levels, β-actin mRNA was determined, and data were expressed as ratios relative to β-actin levels. The sequences of primers were listed in Table 1.

**Table 1:** List of primers used for real-time RT-PCR.

Gene Name	real-time RT-PCR Primer, 5′ to 3′	
	Forward	Reverse
HIF-1α	CACTGCACAGGCCACATTCAT	AAGCAGGTCATAGGCGGTTTC
CXCR4	TCCGTGGCTGACCTCCTCTT	CAGCTTCCTCGGCCTCTGGC
VEGF	GCACTGGACCCTGGCTTT	CGGGGTACTCCTGGAAGATG
IGF-1	GGCATTGTGGATGAGTGTTG	ACGTGGCATTTTCTGTTCCT
HGF	CTCCTCCTGCTTCCTGTCAC	CCCTTGTTTCTGATGCACCT
bFGF	CGACCCACACGTCAAACTA	CCAGGCGTTAAAGAAGAAA
β-actin	GATTACTGCCCTGGCTCCTA	TCATCGTACTCCTGCTTGCT

### Renal Cytokine and Growth Factor Protein Levels

Renal cortical homogenate VEGF, bFGF, HGF and IGF-1 protein levels were determined using ELISA (R&D Systems, Minneapolis, MN, USA). Each ELISA was performed according to the manufacturer’s instructions. Final results were expressed as pg of VEGF, bFGF, HGF or IGF-1 per mg protein.

### Statistical Analysis

Data are expressed as mean ± SD. All analyses were performed using Stata Software (version 10.0). Differences among three or more groups were evaluated using one-way ANOVA, followed by Bonferroni test. The Kruskal-Wallis ANOVA on ranks was used for non-normally distributed data. A *P* value<0.05 was considered statistically significant.

## Supporting Information

Figure S1 ***In vitro*** **assessment of MSC labeled with SPIO or CM-DiI.** Micrographs from (A) Prussian blue staining (Magnification×200) and (B) fluorescent microscopy (Magnification×100) showed strong labeling for both cell tracking markers.
(TIF)

Figure S2 **Effect of CoCl_2_ on the viable of MSC depends on the exposure concentrations and durations.** (A) MSC proliferation was evaluated with MTT analysis. Cell growth inhibition rate of MSC after exposure to CoCl_2_ increased with concentrations beyond 200 µmol/L (*P*<0.05), but didn’t differ between cells treated with concentrations less than 200 µmol/L (*P*>0.05). In addition, this the growth inhibition rate increased within each CoCl_2_ concentration at exposure times greater than 24 h, but this rate was not increased in with exposure times less than 24 h (*P*>0.05). (B) Cell apoptosis was measured by flow cytometry. The concentration of 200 µmol/L and the duration of 24 h were also the same inflection points of MSC apoptosis after exposure to CoCl_2_ as the one in MTT analysis.
(TIF)
