# Supplementary figures and images for: Hypoxic Preconditioning with Cobalt of Bone Marrow Mesenchymal Stem Cells Improves Cell Migration and Enhances Therapy for Treatment of Ischemic Acute Kidney Injury
Source: PLoS One. 2013 May 9;8(5):e62703. doi: 10.1371/journal.pone.0062703 (PMC3650042; doi:10.1371/journal.pone.0062703)

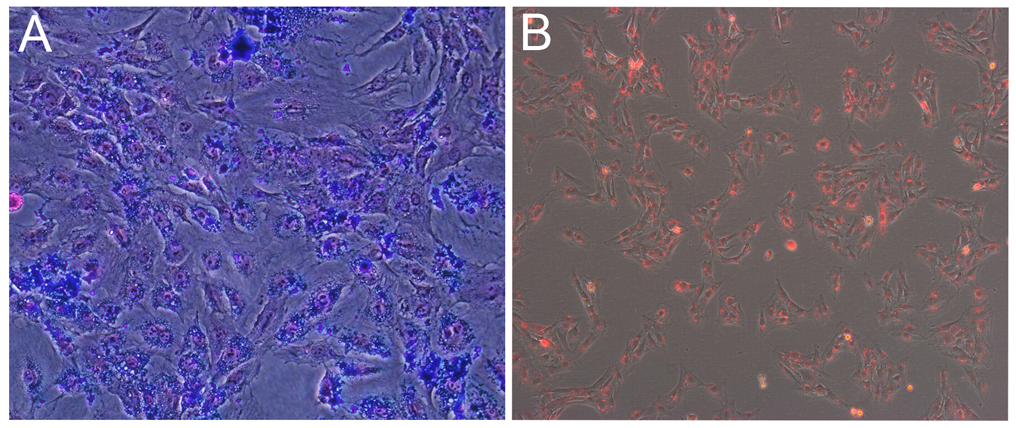

Supplement: Figure S1 — In vitro assessment of MSC labeled with SPIO or CM-DiI. Micrographs from (A) Prussian blue staining (Magnification×200) and (B) fluorescent microscopy (Magnification×100) showed strong labeling for both cell tracking markers. (TIF) [file pone.0062703.s001.tif]

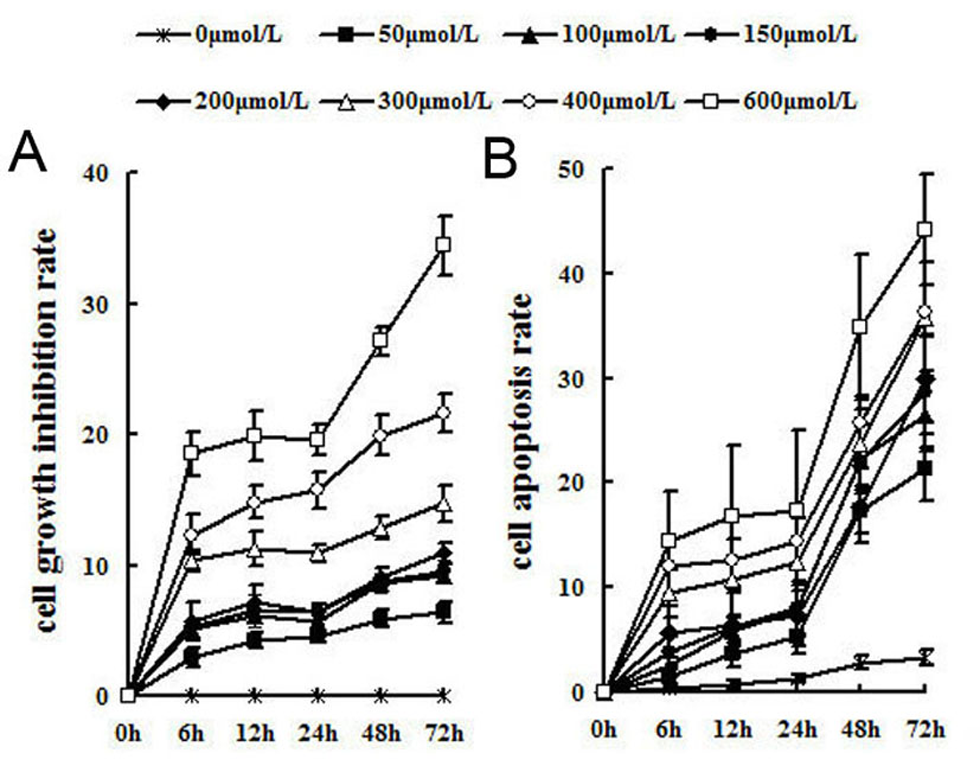

Supplement: Figure S2 — Effect of CoCl2 on the viable of MSC depends on the exposure concentrations and durations. (A) MSC proliferation was evaluated with MTT analysis. Cell growth inhibition rate of MSC after exposure to CoCl2 increased with concentrations beyond 200 µmol/L (P<0.05), but didn’t differ between cells treated with concentrations less than 200 µmol/L (P>0.05). In addition, this the growth inhibition rate increased within each CoCl2 concentration at exposure times greater than 24 h, but this rate was not increased in with exposure times less than 24 h (P>0.05). (B) Cell apoptosis was measured by flow cytometry. The concentration of 200 µmol/L and the duration of 24 h were also the same inflection points of MSC apoptosis after exposure to CoCl2 as the one in MTT analysis. (TIF) [file pone.0062703.s002.tif]
